# Omics Studies for the Identification of Ascidian Peptides, Cognate Receptors, and Their Relevant Roles in Ovarian Follicular Development

**DOI:** 10.3389/fendo.2022.858885

**Published:** 2022-03-07

**Authors:** Tsuyoshi Kawada, Tomohiro Osugi, Shin Matsubara, Tsubasa Sakai, Akira Shiraishi, Tatsuya Yamamoto, Honoo Satake

**Affiliations:** Bioorganic Research Institute, Suntory Foundation for Life Sciences, Kyoto, Japan

**Keywords:** ascidian, *Ciona intestinalis*, peptidomics, transcriptome, peptide, receptor

## Abstract

Omics studies contribute to the elucidation of genomes and profiles of gene expression. In the ascidian *Ciona intestinalis* Type A (*Ciona robusta*), mass spectrometry (MS)-based peptidomic studies have detected numerous *Ciona*-specific (nonhomologous) neuropeptides as well as *Ciona* homologs of typical vertebrate neuropeptides and hypothalamic peptide hormones. Candidates for cognate G protein-coupled receptors (GPCRs) for these peptides have been found in the *Ciona* transcriptome by two ways. First, *Ciona* homologous GPCRs of vertebrate counterparts have been detected by sequence homology searches of cognate transcriptomes. Second, the transcriptome-derived GPCR candidates have been used for machine learning-based systematic prediction of interactions not only between *Ciona* homologous peptides and GPCRs but also between novel *Ciona* peptides and GPCRs. These data have ultimately led to experimental evidence for various *Ciona* peptide-GPCR interactions. Comparative transcriptomics between the wildtype and *Ciona vasopressin* (CiVP) gene-edited *Ciona* provide clues to the biological functions of CiVP in ovarian follicular development and whole body growth. Furthermore, the transcriptomes of follicles treated with peptides, such as *Ciona* tachykinin and cionin (a *Ciona* cholecystokinin homolog), have revealed key regulatory genes for *Ciona* follicle growth, maturation, and ovulation, eventually leading to the verification of essential and novel molecular mechanisms underlying these biological events. These findings indicate that omics studies, combined with artificial intelligence and single-cell technologies, pave the way for investigating in greater details the nervous, neuroendocrine, and endocrine systems of ascidians and the molecular and functional evolution and diversity of peptidergic regulatory networks throughout chordates.

## Introduction

Ascidians are aquatic organisms that live all over the world and belong to the phylum Urochordata and superphylum Chordata; they are one of the closest relatives of vertebrates (1–3). They have been used in developmental biology as model organisms for more than 50 years, and the genome of *Ciona intestinalis* Type A (*Ciona robusta*) was sequenced in 2002 (4). At the dawn of the omics era, expressed sequence tags (ESTs) and gene model analyses identified approximately 16,000 protein-coding genes and various vertebrate orthologs in the *C. robusta* genome (4, 5). Furthermore, the gene expression profiles during embryonic development have been determined by *in situ* hybridization and transcriptomics (6–9), and similarities between the transcriptional network of *C. robusta* and vertebrates have been clarified (10–13). These genomic and transcriptomic studies have highlighted the importance of *C. robusta* in comparative and evolutionary developmental and genome biology (13–16). In contrast, less attention was paid to the reproductive mechanisms of *Ciona*.

In vertebrates, ovarian follicular development is accurately regulated by the hypothalamic-pituitary-gonadal axis (HPG axis). A hypothalamic neuropeptide, gonadotropin-releasing hormone (GnRH), stimulates the synthesis and secretion of gonadotropins in the pituitary, which induces follicle growth, maturation, ovulation, and the production of sex steroid hormones for negative feedback regulation in the ovary (17–20). Recent studies have further revealed that more neuropeptides, including kisspeptin, neurokinin B, dynorphin A, and gonadotropin-inhibitory hormone, play important roles in the regulation of the HPG axis (21, 22). Thus, neuropeptides, as major signaling factors, are responsible for the regulation of the HPG axis in vertebrates. In contrast, *C. robusta* is not endowed with the HPG axis, leading to the presumption that neuropeptides produced in the neuroendocrine system directly regulate follicle development *via* neural network transport instead of regulation by the HPG axis. Combined with the crucial phylogenetic position of ascidians as a sister group of the vertebrates, elucidation of the mechanisms of reproduction regulated by ascidian neuropeptides will provide insight into the molecular and functional evolution of the endocrine and neuroendocrine systems in chordates.

**Figure 1:**
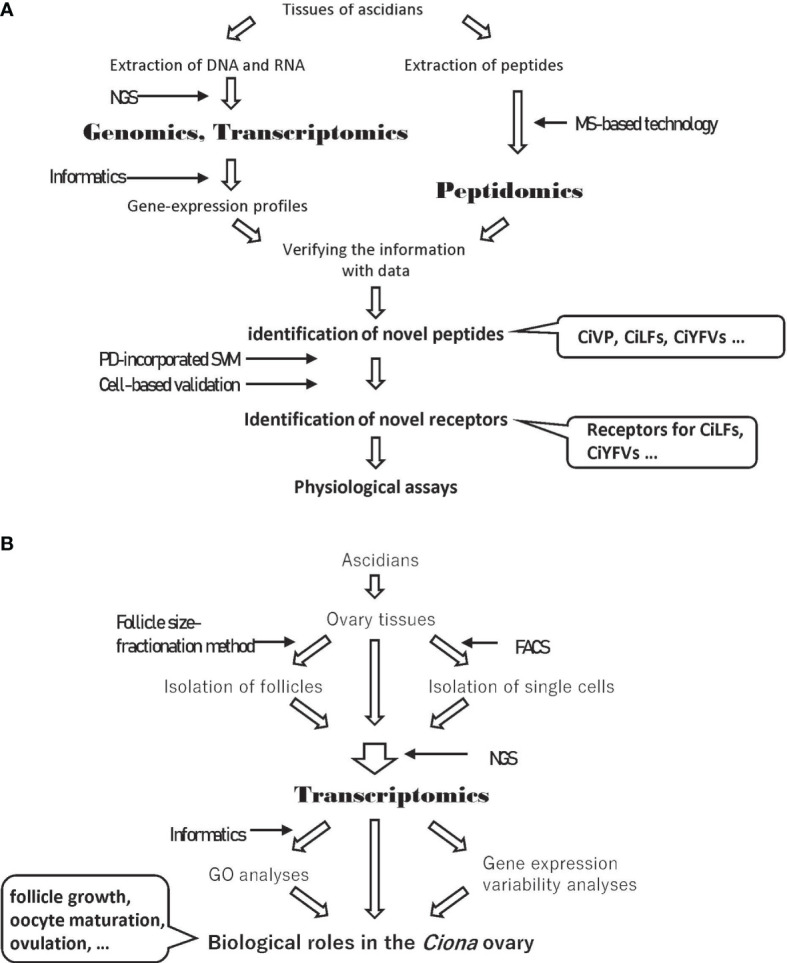
Experimental strategies including omics analyses. **(A)** Experimental strategy to identify novel ascidian peptides and their receptors. **(B)** Experimental strategy to elucidate the biology of the *Ciona* ovary using transcriptome data. NGS, Next Generation Sequencing; MS, Mass Spectrometry; PD, Peptide Descriptor; SVM, Support Vector Machine; FACS, Fluorescence-Activated Cell Sorting; GO, Gene Ontology.

As stated above, gene model analyses of *C. robusta* have found approximately 16,000 protein-coding genes. However, few neuropeptide genes were annotated on the original version, given that homology-based searching methods are frequently useless for the detection of small peptides or their genes due to far less sequence homology information, compared with that of proteins. Over the last two decades, peptidomic analyses have advanced due to mass spectrometry (MS)-based technology, leading to the sensitive detection of small amounts of peptides. An MS-based peptidomic analysis has characterized 33 peptides, including 26 novel peptides, from the *Ciona* neural complex followed by the detection of their genes by referencing the resultant peptide sequences to the genome/EST database and by localization of their gene expression (**Figure 1A**) (23). These ascidian neuropeptides are largely classified into two groups: (i) homologs of vertebrate peptides, and (ii) *Ciona*-specific peptides. The former group includes typical vertebrate neuropeptide homologs, including a vasopressin homolog (CiVP) (24), a tachykinin homolog (CiTK) (25), GnRH homologs (tGnRHs) (26), and a cholecystokinin/gastrin homolog (cionin) (27, 28), while the latter group contains CiLFs, CiYFVs, and CiNTLPs including partial neurotensin-like sequences (23) (**Supplementary Table 1**). The identification of these neuropeptides has paved the way to understanding the ascidian neuroendocrine and nervous systems involved in reproductive functions.

Omics analyses are also powerful tools to identify comprehensive gene expression profiles. Gene ontology (GO) analyses using comprehensive gene expression profiles provide clues to the elucidation of the biological features and functions of target tissues or cells (**Figure 1B**). Comparisons of gene expression profiles between tissues treated or untreated with a target peptide or between target gene-knockdown organisms and wildtype organisms can identify key molecules induced by a target peptide (**Figure 1B**). In this review, we provide an overview of essential neuropeptidergic regulatory mechanisms underlying *Ciona* follicle growth, oocyte maturation, and ovulation, which have been elucidated by a combination of omics and physiological analyses.

## Nervous System and Ovary in *CIONA*

**Figure 2:**
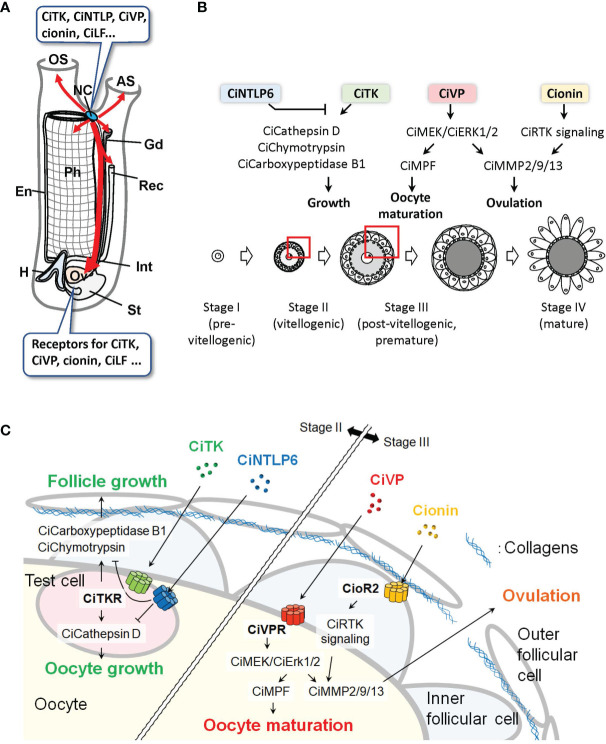
Peptidergic systems in *Ciona*. **(A)** Various neuropeptides are expressed in the neural complex. Peptidergic nerves directly innervate from the neural complex to the peripheral organs (red arrows). The ovary, which expresses various peptide receptors, is a major target of the neuropeptides. AS, atrial siphon; En, endostyle; Gd, gonoduct; H, heart; Int, intestine; NC, neural complex; OS, oral siphon; Ov, ovary; Ph, pharynx; Rec, rectum; St, stomach. **(B, C)** Peptidergic regulation of ovarian follicular growth, oocyte maturation, and ovulation in *Ciona*. Overview **(B)** and the enlarged structure of a follicle **(C)** as indicated by red boxes in **(B)** are shown. In vitellogenic (stage II) follicles, CiTK induces the expression of CiCathepsin D in test cells and CiChymotrypsin and CiCarboxypeptidase B1 in inner follicular cells and promotes follicle growth from stage II to stage III. CiNTLP6 suppresses CiTK-induced gene expression and subsequent follicle growth. In postvitellogenic (stage III) follicles, CiVP activates CiMEK/CiErk1/2 in oocytes and promotes oocyte maturation and ovulation *via* activation of CiMPF and CiMMP2/9/13, respectively. Cionin also induces CiMMP2/9/13 expression *via* CioR2 in inner follicular cells and RTK signaling and subsequent ovulation in stage III follicles.

Recent morphological studies using prohormone convertase 2 promoter-Kaede transgenic *Ciona* showed that the peptidergic nervous system directly innervates to the ovary (29, 30). The innervations of nerves to the ovary were also found in CiVP promoter-Venus transgenic *Ciona*, suggesting that CiVP acts as an endogenous factor in the ovary (31). In addition to these morphological characteristics of the peptidergic nervous system, expression of the receptors for neuropeptides, including CiTK, CiVP, and cionin, was detected in the ovary (24, 25, 32). These findings indicate that the ovary is a major target of the neuropeptides produced in the neural complex of *Ciona* (**Figure 2A**).

*Ciona* ovarian follicles are classified into four major developmental stages: stage I (pre-vitellogenic), stage II (vitellogenic), stage III (post-vitellogenic and pre-mature), and stage IV (mature) oocytes (33). This classification is based on criteria including size, pigmentation of the oocytes, and changes in the cellular layer surrounding the oocytes (33). Recently, an efficient follicle size-fractionation method using stainless steel sieves of various particle sizes was developed (34, 35). This method enables physiological assays as well as transcriptome analyses of the respective stages of follicles. Furthermore, an *in vitro* evaluation of follicle growth, maturation, and ovulation of *Ciona* follicles has been established (34–37). These novel experimental procedures can lead to the investigation of the specific biological functions of neuropeptides on follicles at individual stages.

## Ovarian Follicular Development

Consistent with transcriptomic data showing high expression of the *Citk receptor* gene during follicle development (34), our *in vitro* incubation system and morphological evaluation of isolated follicles demonstrated that CiTK stimulates follicle growth from stage II to stage III (36). Microarray and biochemical analyses of CiTK-treated and -untreated follicles revealed that CiTK induces the expression and enzymatic activities of chymotrypsin, carboxy-peptidase B1, and cathepsin D (36). Moreover, CiTK was shown to directly induce the gene expression and resulting enzymatic activity of cathepsin D in test cells (oocyte-accessory cells) expressing the CiTK receptor, and to secondarily activate the gene expression and resulting enzymatic activity of chymotrypsin and carboxypeptidase B1 in follicle cells (37). These proteases are all responsible for CiTK-induced follicle growth, given that inhibition of any one of these proteases resulted in the arrest of follicle growth (36). Also of interest is that CiNTLP6 treatment for stage II follicles suppresses CiTK-induced gene expression of these proteases and the subsequent follicle development to stage III (23). Consequently, CiTK and CiNTLP6 regulate follicle development from stage II to stage III *via* activation of the gene expression and enzymatic activities of cathepsin D in test cells and chymotrypsin and carboxypeptidase B1 in follicle cells (**Figures 2B, C**) (23, 36, 37). These findings provide evidence that CiTK is essential for the development of premature follicles.

The expression of the CiVP receptor also suggests important roles of CiVP in the ovary (24). In a TALEN-based CiVP mutant of *Ciona*, the number of stage III follicles is markedly decreased and the numbers of stage I and stage II follicles are increased in the ovaries (31). In the ovary of CiVP-mutant *Ciona*, transcriptome analysis and subsequent gene ontology (GO) analysis showed that the downregulated genes were categorized as the genes related to “Binding”, “Phosphorylation”, and “Organelles”, whereas upregulated genes were categorized as the genes related to “Nucleus” or “Ribosomes” (31). Interestingly, the ceramide synthase homologous gene that participates in the removal of low-quality early follicles as a lipid mediator in mammals is downregulated in CiVP-mutants (31). Protein kinase C β, which is an important factor for vesicle transport during the secretion of signaling molecules as well as organelle formation, is also downregulated in CiVP-mutants (31). In contrast, the *MutL-homolog 3* gene, which is known to repair DNA mismatch during DNA replication and meiosis, is upregulated in CiVP-mutants (31). These findings suggest that CiVP plays multiple important roles to maintain the normal growth of early-stage follicles *via* the regulation of diverse genes.

## Oocyte Maturation and Ovulation

In addition to the *in vivo* analysis of CiVP (31), *in vitro* assays and transcriptomic analyses following the aforementioned ovarian follicle fractionation have helped clarify the roles of neuropeptides in *Ciona* oocyte maturation and ovulation (34, 35). In line with receptor expression in the ovary (24, 32), CiVP and cionin have been demonstrated to promote follicle maturation and ovulation *in vitro* (34, 38). Transcriptomic analyses of fractionated follicles have contributed a great deal to our understanding of the essential molecular mechanisms underlying CiVP- and cionin-induced oocyte maturation and ovulation; the expressions of *Civp receptor* and the *Ciona-extracellular signal-related kinase* homolog, *CiErk1/2*, were elevated toward oocyte maturation and ovulation. Gene expression of a matrix metalloproteinase, *CiMmp2/9/13*, was suppressed in MEK (MAPK kinase)-inhibited follicles (i.e., CiErk1/2-inhibited immature, unovulated), compared with those that are uninhibited (mature, ovulated) (34). The expressions of the receptor tyrosine kinase (RTK) signaling genes and *CiMmp2/9/13* were upregulated in cionin-treated follicles, compared with untreated ones (38). Physiological assays and qRT-PCR validation following transcriptomic analyses have verified that CiVP activates CiErk1/2 and promotes oocyte maturation *via* activation of the maturation promoting factor and ovulation *via* induction of CiMmp2/9/13 expression (34), and that cionin also induces CiMmp2/9/13 expression and ovulation *via* upregulation of the RTK signaling genes (38). Combined with visualizing the entire projection of peptidergic neurons from the neural complex to the peripheral tissues (29, 30) and *in vivo* analysis of CiVP (31), *in vitro* physiological analyses have revealed that neuropeptides such as CiVP and cionin are produced in the neural complex (23, 24, 32) and regulate follicular development, oocyte maturation, and ovulation directly through the neuroendocrine system. Consequently, a combination of the classical bioassays and transcriptomic analyses has revealed the major regulatory pathways of *Ciona* oocyte maturation and ovulation (**Figures 2B, C**).

In other invertebrates, species-specific neuropeptides have been identified as maturation-inducing hormones (MIHs) such as W/RPRPamide for jellyfish in the phylum Cnidaria (39), relaxin-like gonad stimulating peptide for starfish (40), and cubifrin (NGIWYamide) for sea cucumber (41) in the phylum Echinodermata. In contrast to the variable MIHs, some molecular mechanisms underlying oocyte maturation and ovulation have been conserved among phyla. For example, the MOS (MAPK kinase kinase)/MAPK-maturation promoting factor (MPF, a complex of Cdk1 and Cyclin B) pathway in oocyte maturation is conserved in jellyfish and vertebrates (42). Moreover, degradation of extracellular matrix by matrix metalloproteinases (MMPs) was also found to be important for ovulation in fish (Vertebrata) (43, 44) and in *Drosophila* (Arthropod) (45). Furthermore, vasotocin, a non-mammalian vertebrate VP family peptide, has been reported to induce oocyte maturation and ovulation in catfish (Vertebrata) (46, 47), and RTK signaling was also reported to be involved in ovulation in mouse (Vertebrata) (48). Collectively, these studies, combined with the aforementioned *Ciona* (Urochordata) oocyte maturation and ovulation processes, shed light on species-specificity of triggering peptides in invertebrates and on the conserved mechanisms of oocyte maturation and ovulation among phyla. Further studies on oocyte maturation and ovulation in various animal species will pave the way for understanding the evolutionary processes of these biological events.

## Conclusion and Perspectives

Omics studies are powerful tools to reveal the molecular mechanisms of biological events. In studies on *Ciona*, peptidomics and transcriptomics and subsequent molecular and physiological analyses have revealed that neuropeptides, including CiTK, CiNTLP6, CiVP, and cionin, regulate follicle growth, oocyte maturation, and ovulation (**Figures 1**, **2B, C**). In addition to omics, newly developed techniques including follicle fractionation and gene-knockout have also contributed to the analysis of signaling networks induced by neuropeptides (**Figure 1B**). Moreover, a comparison of gene expression profiles between the tissues treated or untreated with a target peptide or between target gene-knockdown organisms and wildtype organisms will lead to the elucidation of reliable signaling networks induced by the target peptide (**Figure 1B**). Furthermore, comparisons of transcriptomic profiles of target peptide receptor homolog-expressing cells in closely related species will elucidate the evolution and diversification of signaling networks induced by the target peptide.

To develop omics-based biology to the next stage, single-cell transcriptomes, whose expression profiles exhibit more relevant signaling networks than whole-tissue transcriptomes, will play important roles. In *C. robusta*, Horie et al. analyzed a single-cell transcriptomic profile of the ascidian embryo at the mid-tailbud stage and determined the regulatory mechanism for dopaminergic neurons on the basis of marker gene homolog expression (49). The transcriptome profile showed that the *Pancreas associated transcription factor 1a* (*Ptf1a)* homolog gene is specifically expressed in ascidian dopaminergic neuron cells, and knock-down and misexpression of the *Ptf1a* homolog gene suggest that it functions as a crucial factor in dopaminergic neuronal differentiation (49). Thus, the profiling of single-cell transcriptomic data is a useful method to find crucial factors for biological functions. Moreover, fluorescence-activated cell sorting (FACS) is also a useful technique that can collect single cells expressing a target protein, including receptors of neuropeptides on the plasma membrane. FACS sorting and subsequent transcriptomic analyses of single cells expressing a target receptor provide clues that clarify the effects of the target peptide, uncovering the mechanism of signal transduction *via* peptide-receptor interactions in target cells (**Figure 1B**).

*C. robusta* possesses neuropeptides that are categorized as homologs of mammalian peptides or *Ciona*-specific peptides. Homology-based searches have contributed to the identification of ascidian peptides and receptors of mammalian homologs. However, homology-based identification of receptors for *Ciona*-specific peptides is virtually impossible due to the low sequence identity of *Ciona*-specific peptides or their receptors to known ones. In mammals, ligand-screening of orphan receptors has been widely employed by high-throughput reverse-pharmacological assays based on evaluating typical signal transductions. Due to the time, cost, and labor required, only a limited number of ligand-receptor pairs have been identified recently (50). During the past decade, artificial intelligence (AI) technologies have been developed and utilized to search for ligand-receptor pairs within enormous amounts of omics data (51–53). In a machine-learning system, chemical, physicochemical, and biochemical properties and sequence information of “known” ligand-receptor pairs are converted to numerical vectors (descriptors) and used to predict “novel” ligand-receptor pairs. Recently, the peptide descriptor (PD)-incorporated support vector machine (SVM) was developed to predict novel neuropeptide-receptor interactions, and receptors for *Ciona*-specific peptides were predicted (54). The predicted receptors for *Ciona*-specific peptides, CiLF1, CiLF2, CiLF5, CiLF6, CiLF7, CiLF8, CiYFV1, CiYFV3, and CiNTLP2, were validated by intracellular calcium mobilization assays (**Figure 1A**) (54). Thus, AI-based prediction of ligand-receptor pairs without sequence homology and molecular phylogenetic relatedness is contributing to the deorphanization of receptors. In other words, these studies obviously demonstrate the usefulness of omics data in “post-omics” research strategies, such as the machine learning-based prediction of novel peptide-receptor pairs. CiLF receptors are expressed in the ovaries (54), suggesting that at least several CiLFs participate in ovarian events, including follicle growth, oocyte maturation, or ovulation. In addition, transcriptomic profiles of *Ciona* follicles suggest that several orphan receptors are expressed in the follicles (34). The determination of novel peptide-receptor pairs using PD-incorporated SVM and cell-based validations will surely lead to the verification of the biological events in the ascidian ovary.

Taken together, the integration of omics analyses, physiological analyses, single-cell technologies, and AI technologies will provide novel insight into the mechanisms of the endocrine system and signaling networks in *Ciona*.

## Author Contributions

TK, TO, SM, and HS contributed to the conception and design of the manuscript. TK, TO, SM, TY and HS wrote sections of the manuscript. TK, TO, SM, TS, AS, and HS designed the figures. All authors approved the submitted version.

## Funding

This work was supported by grants from the Japan Society for the Promotion of Science (http://www.jsps.go.jp/english/index.html) to TK (JP20K06752), SM (JP19K16182), and HS (JP19K06752).

## Conflict of Interest

The authors declare that the research was conducted in the absence of any commercial or financial relationships that could be construed as a potential conflict of interest.

## Publisher’s Note

All claims expressed in this article are solely those of the authors and do not necessarily represent those of their affiliated organizations, or those of the publisher, the editors and the reviewers. Any product that may be evaluated in this article, or claim that may be made by its manufacturer, is not guaranteed or endorsed by the publisher.
