# Supplementary material for: Omics Studies for the Identification of Ascidian Peptides, Cognate Receptors, and Their Relevant Roles in Ovarian Follicular Development
Source: Front Endocrinol (Lausanne). 2022 Mar 7;13:858885. doi: 10.3389/fendo.2022.858885 (PMC8936170; doi:10.3389/fendo.2022.858885)
Supplement: Supplementary file 1 [file Table_1.docx]

Supplementary Information

**Supplementary Table 1**

Sequences of *Ciona*-specific peptides. Conserved sequences in each family peptide are shadowed.

| **Peptide** | **Sequence** | **Gene (Accession Number in**  **DDBJ/ENA/GenBank)** |
| --- | --- | --- |
| CiNTLP1 | pQLHVPSIL | *ci-nt-A* (BR000876) |
| CiNTLP2 | GMMGPSII | *ci-nt-A* (BR000876) |
| CiNTLP3 | MMLGPGIL | *ci-nt-A* (BR000876) |
| CiNTLP4 | FGMIPSII | *ci-nt-A* (BR000876) |
| CiNTLP5 | NKLLYPSVI | *ci-nt-B* (BR000879) |
| CiNTLP6 | SRHPKLYFPGIV | *ci-nt-B* (BR000879) |
|  |  |  |
| CiLF1 | FQSLF | *ci-lf* (BR000881) |
| CiLF2 | YPGFQGLF | *ci-lf* (BR000881) |
| CiLF3 | HNPHLPDLF | *ci-lf* (BR000881) |
| CiLF4 | YNSMGLF | *ci-lf* (BR000881) |
| CiLF5 | SPGMLGLF | *ci-lf* (BR000881) |
| CiLF6 | SDARLQGLF | *ci-lf* (BR000881) |
| CiLF7 | YPNFQGLF | *ci-lf* (BR000881) |
| CiLF8 | GNLHSLF | *ci-lf* (BR000881) |
|  |  |  |
| CiYFV1 | ELVVRDPYFV | *ci-yfv/l* (BR000885) |
| CiYFV2 | NNQESYFV | *ci-yfv/l* (BR000885) |
| CiYFV3 | DDEPRSYFV | *ci-yfv/l* (BR000885) |
| CiYFL1 | DAARPNYYFL | *ci-yfv/l* (BR000885) |
